# Supplementary material for: The Impact of Jujube Witches’ Broom Phytoplasma on the Community Structure of Endophytes in Jujube
Source: Microorganisms. 2025 Jun 12;13(6):1371. doi: 10.3390/microorganisms13061371 (PMC12196086; doi:10.3390/microorganisms13061371)
Supplement: Supplementary file 1 [file microorganisms-13-01371-s001.zip › microorganisms-3636890-supplementary.pdf]

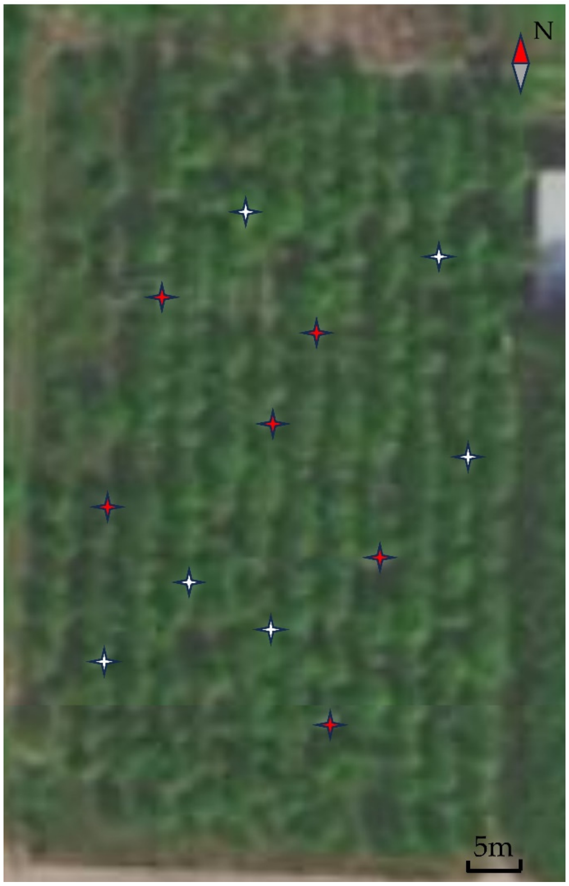

Figure S1. Spatial distribution of sampling trees in the orchard

The satellite map of the orchard is screenshot from http://map.baidu.com at 24/05/2025

✧ represents healthy jujube trees; ✦ represents diseased jujube trees.

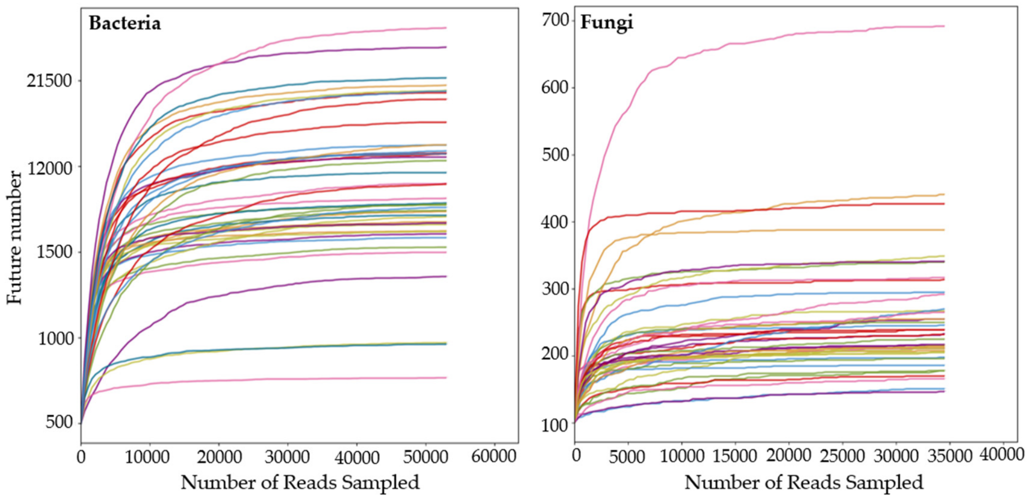

Figure S2. Species dilution curve of the endophytic bacteria and fungi

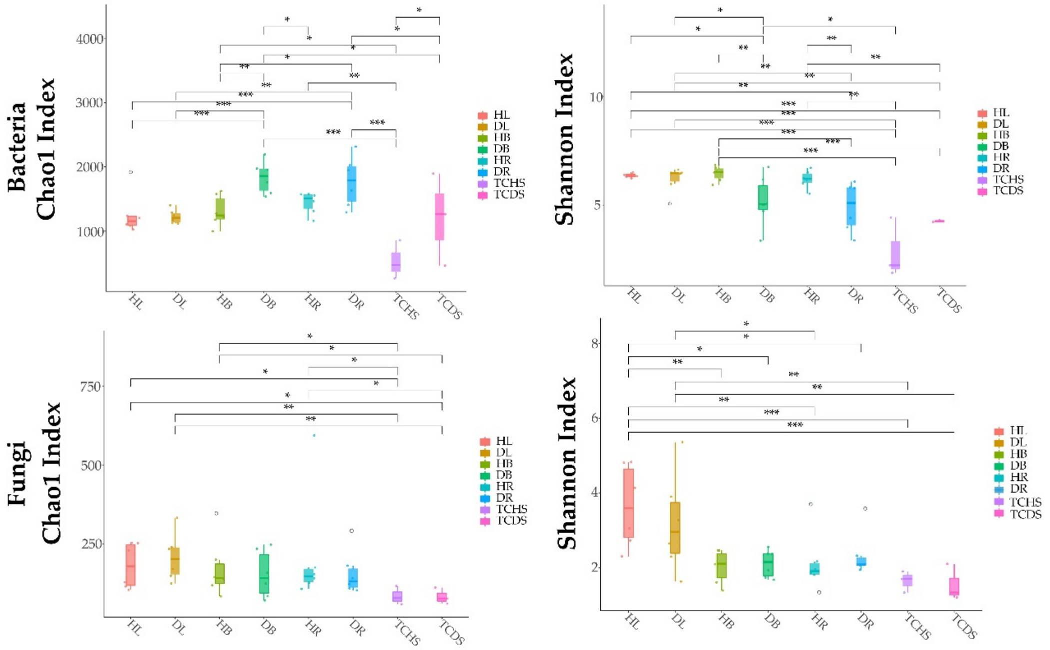

Figure S3. α diversity analysis of the endophytic bacteria and fungi

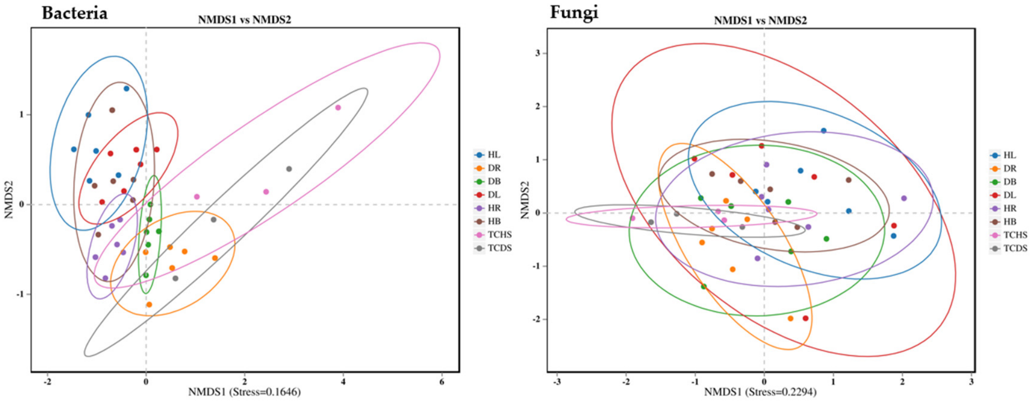

Figure S4. β diversity analysis of the endophytic bacteria and fungi

Table S1. Sample collection information

Sample name	Compartment	Health condition	Duplicate quantity
HL	Leaf	Healthy	6(HL1-HL6)
DL	Leaf	Diseased	6(DL1-DL6)
HB	Branch	Healthy	6(HB1-HB6)
DB	Branch	Diseased	6(DB1-DB6)
HR	Root	Healthy	6(HR1-HR6)
DR	Root	Diseased	6(DR1-DR6)
TCHS	Whole plant	Healthy	3(TCHS1-TCHS3)
TCDS	Whole plant	Diseased	3(TCDS1-TCDS3)

Table S2. Primer information

Type	Amplified region	Primer name	Primer sequence
Bacterial	16sv3+v4	338F	5'- ACTCCTACGGGAGGCAGCA-3
amplification [1]	16sv3+v4	806R	5'- GGACTACHVGGGTWTCTAAT-3
Fungal	ITS1	ITS1	5'-CTTGGTCATTTAGAGGAAGTAA-3
amplification [2]	ITS1	ITS2	5'-GCTGCGTTCTTCATCGATGC-3'
Phytoplasma		U3	5'-TTCAGCTACTCTTTGTAACA-3'
detection [3]		U5	5'-CGGCAATGGAGGAAACT-3'

1. Hong, C.; Si, Y.; Xing, Y.; Li, Y. Illumina MiSeq sequencing investigation on the contrasting soil bacterial community structures in different iron mining areas. *Environmental Science and Pollution Research* 2015, 22, 10788-10799, doi:10.1007/s11356-015-4186-3.

2. Orgiazzi, A.; Lumini, E.; Nilsson, R.H.; Girlanda, M.; Vizzini, A.; Bonfante, P.; Bianciotto, V. Unravelling soil fungal communities from different Mediterranean land-use backgrounds. *PloS One* 2012, 7, e34847, doi:10.1371/journal.pone.0034847.

3. Lorenz, K.H.; Schneider, B.; Ahrens, U.; Seemuller, E. Detection of the apple proliferation and pear decline phytoplasmas by PCR amplification of ribosomal and nonribosomal DNA. *Phytopathology*, 1995, 85(7): 771-776.
